# Alleviation of calcium hydroxide nanoparticles induced genotoxicity and gastritis by coadministration of calcium titanate and yttrium oxide nanoparticles in mice

**DOI:** 10.1038/s41598-023-49303-x

**Published:** 2023-12-12

**Authors:** Hanan R. H. Mohamed, Salma H. Elbasiouni, Ahmed H. Farouk, Kirolls A. Nasif, Karima Nasraldin, Gehan Safwat

**Affiliations:** 1 Zoology Department Faculty of Science, Cairo University, Giza, Egypt; 2 Faculty of Biotechnology, October University for Modern Sciences and Arts, 6th of October City, Egypt

**Keywords:** Molecular biology, Risk factors

## Abstract

Diverse applications of nanoparticles due to their unique properties has rapidly increased human exposure to numerous nanoparticles such as calcium hydroxide (Ca(OH)_2_), calcium titanate (CaTiO_3_), and yttrium oxide (Y_2_O_3_) nanoparticles almost in all aspect of daily life. However, very limited data are available on the effect of these nanoparticles on genomic DNA integrity and inflammation induction in the gastric tissues. Hence, this study estimated the effect of Ca(OH)_2_, CaTiO_3,_ or/and Y_2_O_3_ nanoparticles multiple oral administration on the genomic DNA damage and inflammation induction in the mice gastric tissues. A suspension containing 50 mg/kg b.w of Ca(OH)_2_, CaTiO_3,_ or Y_2_O_3_ nanoparticles were given orally to male mice separately or together simultaneously three times a week for two consecutive weeks. Multiple oral administration of Ca(OH)_2_ nanoparticles led to significant elevations in DNA damage induction and ROS generation, in contrast to the non-significant changes observed in the level of induced DNA damage and generated ROS after administration of CaTiO_3_ or Y_2_O_3_ nanoparticles separately or in combination with Ca(OH)_2_ nanoparticles. Oral administration of Ca(OH)_2_ nanoparticles alone also highly upregulated INOS and COX-2 genes expression and extremely decreased eNOS gene expression. However, high elevations in eNOS gene expression were detected after multiple administration of CaTiO_3_ and Y_2_O_3_ nanoparticles separately or together simultaneously with Ca(OH)_2_ nanoparticles. Meanwhile, non-remarkable changes were noticed in the expression level of INOS and COX-2 genes after administration of CaTiO_3_ and Y_2_O_3_ nanoparticles separately or simultaneously together with Ca(OH)_2_ nanoparticles. In conclusion: genomic DNA damage and inflammation induced by administration of Ca(OH)_2_ nanoparticles alone at a dose of 50 mg/kg were mitigated by about 100% when CaTiO_3_ and Y_2_O_3_ nanoparticles were coadministered with Ca(OH)_2_ nanoparticles until they reached the negative control level through altering the expression level of eNOS, INOS and COX-2 genes and scavenging gastric ROS. Therefore, further studies are recommended to investigate the toxicological properties of Ca(OH)_2_, CaTiO_3_ and Y_2_O_3_ nanoparticles and possibility of using CaTiO_3_ and Y_2_O_3_ nanoparticles to mitigate genotoxicity and inflammation induction by Ca(OH)_2_ nanoparticles.

## Introduction

Nanoparticles are materials that range in size from 1 to 100 nm and have novel size-dependent properties that differ from those of the same bulk materials. Thus nanomaterials are used in various applications including agricultural, environmental, biotechnology, waste disposal, industrial applications, and many other products e.g. electronics, clothes, optics, cosmetics, food, engineering and medicine^1–3^.

For example, nanoparticles of calcium hydroxide (Ca(OH)_2_) or slaked lime, are an inorganic chemical compound, widely used in various applications especially in food processing, wastewater treatment, cement, plastic production and dentistry^4,5^**.** Similarly, calcium titanate (CaTiO_3_) nanoparticles, a member of the metal titanate family, have sparked ongoing interest in both basic and applied material science and have recently been used in aquatic habitats to decompose contaminants, and are also used as a biomaterial to deliver calcium ions for osteoblasts differentiation, mineralization of bone marrow stem cells, and cells’ proliferation^6,7^**.** In addition, inorganic yttrium oxide (Y_2_O_3_) nanoparticles are a type of precious rare earth element that appears as a white powder and is frequently used in the production of microwave filters, cathode ray display panels, and plasma televisions, as an additive in paint, plastic and steel, as well as in biomedical applications including drug delivery, biosensors, bio-imaging, fluorescence imaging, and cancer treatments^8,9^.

All of these uses increase the risk of human intake of Ca(OH)_2_, Ca(OH)_2_ and Y_2_O_3_ nanoparticles together through contaminated food and water and medications. However, the ingestion extent and the potential risks posed by these nanoparticles exposure remain poorly studied, but the physical and chemical properties of nanoparticles are strongly influenced by their local microenvironment. The stomach is the most advanced and developed endocrine gland and the stomach diseases and their degree of severity vary. For example, gastritis is a term used to describe inflammation of the gastric mucosa, and can lead to cancer, dysplasia, and metaplasia. In developing nations, chronic gastritis is still a very frequent condition^10^. Consequently, all these necessitated studying the effect of Ca(OH)_2_, Ca(OH)_2_ and Y_2_O_3_ nanoparticles on the integrity of gastric tissues.

However, information is lacking on effect of Ca(OH)_2_, Ca(OH)_2_ and Y_2_O_3_ nanoparticles administration on the integrity of gastric tissues but few recent studies have been conducted on their genotoxicity and inflammation induction. For example, the study of Mohamed and her colleagues^6^ demonstrated the genetic safety of CaTiO_3_ nanoparticles towards normal human skin fibroblast (HSF) cells through the non-remarkable changes observed in the genomic DNA integrity, ROS generation and expression levels of apoptotic genes after HSF cells treatment with CaTiO_3_ nanoparticles. On the other hand, differential genotoxicity, apoptosis, mitochondrial DNA damage and inflammation induction by single oral administration of Ca(OH)_2_ nanoparticles have been demonstrated in various mice tissues: liver, brain, kidney, bone marrow, lung, heart and spleen^11,12^.

Therefore, the current study was done to estimate the possible DNA damage and inflammation induction by multiple oral administration of Ca(OH)_2_, CaTiO_3_, or/and Y_2_O_3_ nanoparticles in the gastric tissue of mice. That was conducted by performing alkaline comet assay to measure the genomic DNA integrity, and 2,7-dichlorofluorscein dye to detect the ROS production within gastric cells, along with quantitative real time PCR to quantify the expression level of COX-2, iNOS, and eNOS genes.

## Materials and methods

### Characterization of the tested nanoparticles

Powders of Ca(OH)_2_ nanoparticles were purchased from Nanotech Company (Giza, Egypt), while CaTiO_3_ and Y_2_O_3_ nanoparticles were purchased from Sigma Aldrich Company (St. Louis, MO, USA) with a particle size less than 100 nm. Characterization of Ca(OH)_2_, CaTiO_3_, and Y_2_O_3_ nanoparticles has been well conducted using transmission electron microscope (TEM), X-ray diffraction (XRD) and dynamic laser scattering (DLS) to confirm the purity and nan-size of dispersed nanoparticles^6,11,13^.

### Animals

Forty five male Swiss webster mice aging 10–12 weeks and weighting 20–25 g were used in this study and were kept in the animal house at Zoology department, Faculty of Science, Cairo University under normal conditions of light/dark cycles and receiving standard diet pellets and water. Mice were left for 1 week before starting administration to be acclimatized with animal house condition.

### Ethical consideration

The plan and experiments of this study were approved by the MSA University Research Ethics Committee. This study was reported according to ARRIVE guidelines and also Animal handling and experimentations were conducted in accordance with the Guidelines of the National Institutes of Health (NIH) regarding the care and use of animals for experimental procedures.

### Preparation of tested nanoparticles for administration

Nanopowders of Ca(OH)_2_, CaTiO_3_, or Y_2_O_3_ were weighted and suspended in deionized distilled water to prepare the appropriate dose. Suspension of tested nanoparticles were also ultrasonicated using the biologics ultrasonic homogenizer (Model 150VT) immediately prior to animal administration. For simultaneous administration of the three tested nanoparticles, a suspension containing 50 mg/kg of Ca(OH)_2_, CaTiO_3_, and Y_2_O_3_ nanoparticles with equal ratio 1:1:1 was prepared.

### Determination of the nanoparticles tested doses

An acute toxicity test was done to determine the tested dose of Ca(OH)_2_, CaTiO_2_ and Y_2_O_3_ nanoparticles based on the Organization of Economic Cooperation and Development (OECD) quidelines-420. Twenty male mice were randomly divided into four groups, five mice each: a control group and three treated groups. The mice of the control group were orally given deionized distilled water, while, mice of the treated groups were orally given Ca(OH)_2_, CaTiO_2_ or Y_2_O_3_ nanoparticles at the dose level of 2000 mg/kg b.w. All mice of the four groups were monitored carefully for 24 h after given a single dose of nanoparticles and then observed daily for 14 days for any morphological sign or behavior of toxicity. The tested dose of the three used nanoparticles was calculated based the OECD guidelines-420 (fixed dose method) to be as 2.5% (50 mg/kg b.w) of the safety tested dose^14,15^.

### Experimental design

Twenty five male mice were randomly divided into five groups, with five mice in each group. Mice of group I (negative control) group were orally intake deionized distilled water, while mice of II, III, IV and V groups received oral suspension of Ca(OH)_2_, CaTiO_2_ or/and Y_2_O_3_ nanoparticles three times a week for two consecutive weeks. The mice of the five groups were then sacrificed by cervical dislocation and dissected to obtain stomach tissues which were washed with cold phosphate buffered saline (PBS) and stored at − 80° for further studies.

### Generation of gastric ROS

The level of ROS formation within gastric cells was determined based on the formation of a highly fluorescent compound dichlorofluoroscein as a result of the selective reaction between the 2,7-dichloroflurescin diacetate (DCFH-DA) and intracellular ROS^16^. A small portion of fresh stomach tissue (≈50 mg) was gently minced in cold PBS, then the cell suspension was mixed with DCFH-DA dye (20 mM), the mixture was incubated in the dark for 30 min, and then the cells were spread on slides for visualization under epi-fluorescent microscope at 200× magnification.

### Genotoxicity estimation using alkaline Comet assay

The effect of Ca(OH)_2_, CaTiO_3_, or/and Y_2_O_3_ NPs administration on the integrity of the gastric genomic DNA was studied using the alkaline Comet assay^17^ with minor modifications. The prepared slides were stained ethidium bromide, examined and photographed using an epi-fluorescent microscope at a magnification of 200× and finally fifty Comet nuclei were analyzed and scored using the scoring software TriTek Comet Score™ Freeware v1.5. The three comet parameters: tail length, % DNA in tail, and tail moment were used as an indicator for genomic DNA damage and presented as mean ± SD.

### Analysis of genes expression

The gastric mRNA expression level of cyclooxygenase-2 (COX-2) and endothelial (eNOS) and inducible (iNOS) nitric oxide synthase genes was measured in the studied five groups using Real-time polymerase chain reaction (RT-PCR). To do that total gastric RNA was extracted using Thermo Scientific, USA’s GeneJET RNA Purification Kit, and reversely transcribed into complementary DNA using Revert Aid First Strand cDNA Synthesis Kit (Thermo Scientific, USA). A separate RT-PCR was conducted for each gene using SYBER Green master mix and the previously designed primers shown in Table 1^18–20^ by the 7500 Fast system (Applied Biosystem 7500, Clinilab, Egypt). The expression level of theCOX-2, iNOS and eNOS genes were standardized against expression of the housekeeping ß-actin gene and the comparative Ct (ΔΔCt) method was utilized to calculate the genes expression. Results are displayed as mean ± SD.

**Table 1:** The sequences of the used primers in real time PCR.

Gene	Strand	Sequence
iNOS	Forward	5′-CGGGCATTGCTCCCTTCCGAAAT-3′
	Reverse	5′-CTTCATGATAACGTTTCTGGCTCT-3′
eNOS	Forward	5′-TTCCGGCTGCCACCTGATCCTAA-3′
	Reverse	5′-ACCATATGTCCTTGCTCAAGGCA-3′
COX-2	Forward	5′-ACCATTTGAACTATTCTACCAGC-3′
	Reverse	5′-AGTCGGCCTGGGATGGCATCAG-3′
β-actin	Forward	5′-TCA CCC ACA CTG TGC CCA TCT ACGA-3′
	Reverse	5′-GGA TGC CAC AGG ATT CCA TAC CCA-3′

### Statistical analysis

The obtained results are presented as mean ± SD and were analyzed using the Statistical Package for the Social Sciences (SPSS 20). To determine the influence of Ca(OH)_2_, CaTiO_2_ or/and Y_2_O_3_ nanoparticles oral administration on the induction of DNA damage, intracellular ROS production and gene expression level, One-Way analysis of Variance (ANOVA) followed was carried out. Additionally, the homogenity between the five experimental groups were studied using Duncan's test.

## Results

### Nanoparticles characterization

Nanoparticles of Ca(OH)_2_, CaTiO_3_ and Y_2_O_3_ have been well characterized in our recent published studies^6,11,13^ and the XRD pattern, DLS analysis and TEM imaging revealed the purity of the purchased nanoparticles as well as the stability and well distribution of the suspended nanoparticles in deionized distilled water. The Zeta potential value of − 53.2 mV confirmed the stability and low aggregations of Y_2_O_3_ nanoparticles, while, the low Zeta potential values of dispersed Ca(OH)_2_ (2.48 mV) and CaTiO_3_ (− 3.38 mV) nanoparticles revealed low stability and high aggregations, consequently, suuspension of used nanoparticles were highly sonicated immediately prior administration to ensure well distribution of administered nanoparticles. The TEM imaging of nano-suspensions showed the spherical and polyhedral morphology of Ca(OH)_2_, CaTiO_3_ and Y_2_O_3_ nanoparticles, and also confirmed the well distribution of administered nanoparticles. The average particles' size of Ca(OH)_2_, CaTiO_3_ and Y_2_O_3_ nanoparticles was found to be 15.57, 88.79 and 14.00 nm, respectively^6,11,13^**.**

### The nanoparticles tested dose

No morphological or behavioral sign of toxicity was observed while observing all mice in the control group and the three treated groups orally given 2000 mg/kg b.w of Ca(OH)_2_, CaTiO_3_ or Y_2_O_3_ nanoparticles and all mice were still healthy throughout the experimental period. Thus, the lethal half dose (LD50) of Ca(OH)_2_, CaTiO_3_ and Y_2_O_3_ nanoparticles was considered to be higher 2000 mg/kg according to the OECD-420 guidelines, and the tested dose of the three used nanoparticles in this study was calculated as 2½% (50 mg/kg body weight) of the LD50 Obtained from acute toxicity test.

### Generation of gastric ROS

**Figure 1:**
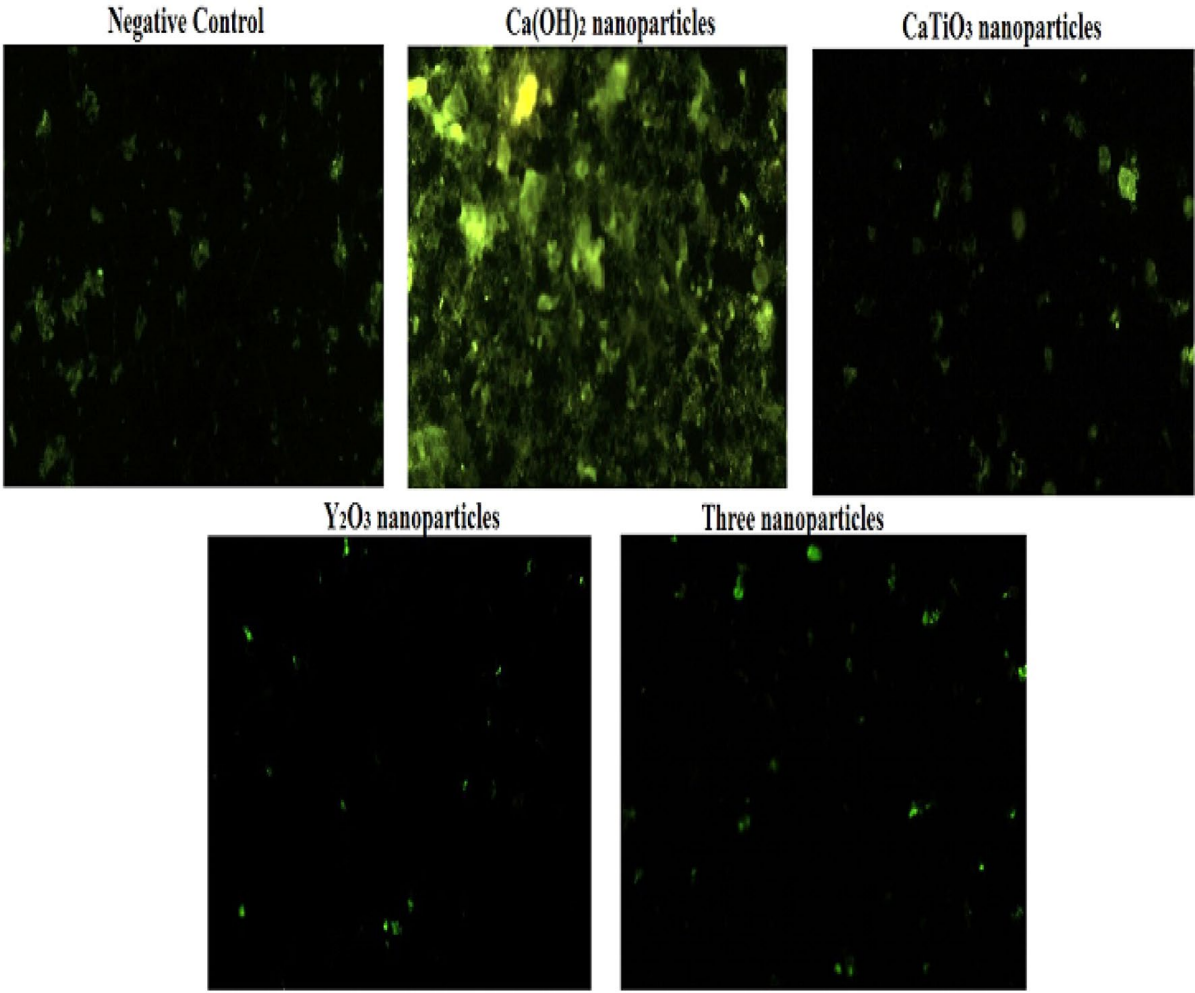
Level of ROS generated within the gastric cells of the negative control group and groups orally administered Ca(OH)_2_, CaTiO_3_ or/and Y_2_O_3_ nanoparticles.

As displayed in Fig. 1 oral administration of Ca(OH)_2_ nanoparticles (Group II) caused dramatic elevation in the generation of ROS within gastric cells, meanwhile, slight elevation was noticed in the ROS generated within gastric cells of mice orally given CaTiO_3_ nanoparticles (Group III) as visualized from the fluorescent green light emitted from the DCFH-DA stained cells compared to that emitted from the stained negative control cells (Group I). On contrary, oral administration of Y_2_O_3_ nanoparticles alone (Group IV) or in combination with Ca(OH)_2_ and nanoparticles (Group V) caused non observable changes in the ROS level generated within gastric cells of mice compared to the negative control ROS level (Fig. 1).

### Genomic DNA instability

**Figure 2:**
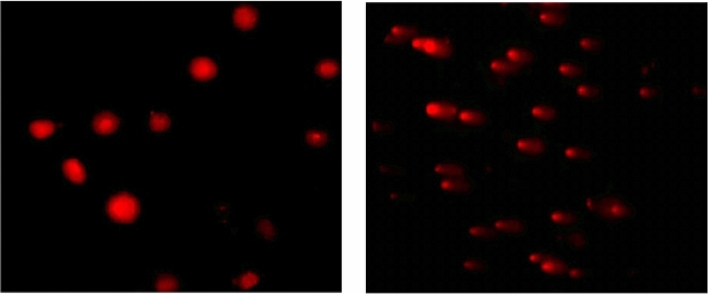
Representative photos for the scored comet intact with intact and damaged DNA regardless treatment.

Results of the alkaline Comet assay revealed a loss of genomic DNA stability in the gastric tissue of mice orally given Ca(OH)_2_ nanoparticles alone (Group II) as manifested by statistically significant elevations in tail length (p < 0.001) and tail moment (p < 0.01) compared to the negative control (Group I) values as seen in Table 2. On the other hand, non-remarkable changes (p > 0.05) in the measured parameters of genomic DNA damage: tail length, %DNA in tail and tail moment were noticed in the gastric tissues of mice orally given CaTiO_3_ (Group III) and Y_2_O_3_ (Group IV) nanoparticles separately or with Ca(OH)_2_ nanoparticles (Group V) as shown in Table 2. Representative examples for the scored comet nuclei with intact and damaged DNA are displayed in Fig. 2.

**Table 2:** Tail length (px), %DNA in tail and tail moment in the gastric tissue of the negative control group and groups administered Ca(OH)_2_, CaTiO_3_ or/and Y_2_O_3_ nanoparticles.

Group	Treatment	Tail length (px)	%DNA in tail	Tail moment
I	Negative control	4.41 ± 1.49^a^	32.35 ± 10.10^a^	1.82 ± 1.21^a^
II	Ca(OH)_2_ nanoparticles	11.97 ± 0.59^b^	35.79 ± 6.59^a^	5.07 ± 1.31^b^
III	CaTiO_3_ nanoparticles	4.95 ± 1.23^a^	26.62 ± 6.98^a^	1.78 ± 0.88^a^
IV	Y_2_O_3_ nanoparticles	3.39 ± 0.29^a^	22.21 ± 4.60^a^	1.24 ± 0.36^a^
V	Three nanoparticles	4.55 ± 1.13^a^	30.95 ± 6.67^a^	1.82 ± 0.65^a^
One way analysis of variance (ANOVA)		F = 31.34 p < 0.001	F = 1.63 p > 0.05	F = 7.89 p < 0.01

Results are expressed as mean ± SD.

Results were analyzed using one-way analysis of variance followed by Duncan’s test to test the similarity between the control and three treated groups.

Means with different letters indicates statistical significant difference between the compared groups in the same column.

### Genes’ expression

Gastric genes expression analysis using RT-PCR demonstrated that multiple oral administration of Ca(OH)_2_ nanoparticles (Group II) caused statistically significant elevations (p < 0.001) in the gastric iNOS and COX-2 genes expression, but it is caused non-significant decrease (p > 0.05) in the iNOS gene expression compared to the negative control expression levels (Table 3). Meanwhile, multiple oral administration of CaTiO_3_ (Group III) and Y_2_O_3_ (Group IV) nanoparticles separately or simultaneously with Ca(OH)_2_ nanoparticles (Group V) caused remarkable high elevations the gastric eNOS gene expression and non-significant changes in the gastric iNOS and COX-genes expression compared to their expression level in the negative control group as shown in Table 3.

**Table 3:** Expression level of eNOS, iNOS and COX-2 genes in the gastrictissue of the negative control group and groups orally administered Ca(OH)_2_, CaTiO_3_ or/and Y_2_O_3_ nanoparticles.

Group	Treatment	eNOS	iNOS	COX-2
I	Negative control	1.00 ± 0.00^a^	1.00 ± 0.00^a^	1.00 ± 0.00^a^
II	Ca(OH)_2_ nanoparticles	0.91 ± 0.12^a^	2.16 ± 0.06^b^	2.06 ± 0.19^b^
III	CaTiO_3_ nanoparticles	1.54 ± 0.16^b^	1.10 ± 0.01^a^	1.12 ± 0.03^a^
IV	Y_2_O_3_ nanoparticles	2.14 ± 0.07^c^	1.00 ± 0.08^a^	0.93 ± 0.05^a^
V	Three nanoparticles	2.39 ± 0.14^d^	1.02 ± 0.06^a^	0.97 ± 0.04^a^
One way analysis of variance (ANOVA)		F = 132.35 p < 0.001	F = 252.01 p < 0.001	F = 81.49 p < 0.001

Results are expressed as mean ± SD.

Results were analyzed using one-way analysis of variance followed by Duncan’s test to test the similarity between the control and three treated groups.

Means with different letters indicates statistical significant difference between the compared groups in the same column.

## Discussion

The exponential increase in the applications and utilization of Ca(OH)_2,_ CaTiO_3_ and Y_2_O_3_ nanoparticles in intensive industrial, medical and food applications highly increase their production and environmental release causing their high accumulation in many environmental compartments such as air, water, and soil and even in food. Consequently, attention has been paid to study the potential risks of these nanoparticles on human health particularly gastrointestinal risks^3^. Unfortunately the biological effects and genotoxicity of orally intake Ca(OH)_2,_ CaTiO_3_ or/and Y_2_O_3_ nanoparticles on gastric tissues are poorly understanding. Therefore, the present study was done to estimate the genomic instability and inflammation induction by Ca(OH)_2,_ CaTiO_3_ or/and Y_2_O_3_ nanoparticles in the gastritis of mice.

In this study Ca(OH)_2,_ CaTiO_3_ and Y_2_O_3_ nanoparticles were orally administered to mice because oral intake of nanoparticles is a main route of Ca(OH)_2,_ CaTiO_3_ and Y_2_O_3_ nanoparticles exposure due their wide uses in food and other products, along with the potential for unintentional ingestion from environmental contamination such as contaminated water and food. Upon oral ingestion, nanoparticles disperse and penetrate the gastric tissues attacking proteins, lipids, carbohydrates and DNA^3,11,12,21^.

The results of the current study demonstrated the non-genotoxic effects of CaTiO_3_ or Y_2_O_3_ nanoparticles through non-significant changes in the tail length, %DNA in tail and tail moment noticed after multiple oral administrations of CaTiO_3_ or Y_2_O_3_ nanoparticles separately. Similarly, recent studies have shown that CaTiO_3_ and Y_2_O_3_ nanoparticles are non-genotoxic on normal human skin fibroblast (HSF) and retinal epithielial-1 (REP1) cells, respectively, through non-remarkable changes in the genomic DNA integrity after cells treatment with CaTiO_3_ or Y_2_O_3_ nanoparticles^6,13^**.**

Based on the results obtained from RTPCR, the demonstrated safety of CaTiO_3_ and Y_2_O_3_ nanoparticles on gastric genomic DNA may result from the marked overexpression of the gastric eNOS gene noticed after administration of CaTiO_3_ or Y_2_O_3_ nanoparticles because overexpression of the eNOS gene maintains the integrity of the gastric tissue by regulating gastric blood flow and stimulating the synthesis and secretion of gastric mucus besides reducing ROS formation^22^. Accordingly, the observed non-significant changes in the gastric ROS generation level and the expression level of the studied pro-inflammatory mediators (iNOS and COX-2 genes) revealed the absence of inflammation and oxidative stress induction after administration of CaTiO_3_ or Y_2_O_3_ nanoparticles and could also be attributed to the above mentioned marked upregulation of gastric eNOS gene expression.

Surveying ROS generation within gastric tissues using 2,7 DCFH-DA demonstrated marked over ROS generation after oral administration of Ca(OH)_2_ nanoparticles that disrupt homeostasis and induce oxidative stress consistent with the reported oxidative stress induction through high ROS generation in mice given orally a single dose of Ca(OH)_2_ nanoparticles^11,12^. High ROS generation and oxidative stress induction unbalance cell membrane permeability and cause many pathological conditions such as genomic DNA damage, neurodegenerative disorders, inflammation, and cancer^6,23,24^.

Induction of genomic DNA damage by chronic Ca(OH)_2_ nanoparticles administration was manifested in this study by the remarkable high elevations in the gastric DNA damage indicating parameters measured by the alkaline Comet assay. Ongoing with the genotoxicity of Ca(OH)_2_ nanoparticles demonstrated in previous studies, this genotoxic effect can be attributed to the aforementioned over-ROS production within the gastric tissues of mice given orally Ca(OH)_2_ nanoparticles alone^11,12^**.**

Inflammation occurs naturally in response to various injurious agents such as the infectious pathogens and toxins. However, persistent exposure to inflammatory agents induces chronic inflammation through disruption of the immune system causing many diseases such as rheumatoid arthritis, chronic gastritis atherosclerosis, diabetes, septic shock, chronic hepatitis and inflammatory neurodegenerative diseases^25^. Once toxins and pathogens induce high ROS generation and DNA damage, inflammation is induced leading to cellular malfunction, excessive secretion of inflammatory cytokines and mediators, tissue degenerative changes and metabolic complications^25,26^.

Inflammation induction after Ca(OH)_2_ nanoparticles administration was demonstrated in this study through the marked elevations observed in the expression level of the gastric iNOS and COX-2 genes and may result from the noticed high ROS generation within the gastric tissue of mice orally administered Ca(OH)_2_ nanoparticles alone. Consistent with previous studies besides high generation of ROS, significant elevations in the iNOS gene expression seen after Ca(OH)_2_ nanoparticles lead to over-production of harmful nitric oxide that reacts with superoxide anions forming reactive nitrogen species (RNS). Accordingly elevations in the ROS and RNS generation stimulate upregulation of the pro-inflammatory mediator COX-2 inducing sever inflammation^12,27,28^.

Extensive uses of Ca(OH)_2_, CaTiO_3_ and Y_2_O_3_ nanoparticles in various applications increase their environmental release and accumulation in contaminated water, soil and food, which together increases the risk of human exposure to these nanoparticles^3^ and necessitates studying the genotoxic effect of simultaneous exposure to Ca(OH)_2,_ CaTiO_3_ and Y_2_O_3_ nanoparticles. Accordingly, the effect of Ca(OH)_2,_ CaTiO_3_ and Y_2_O_3_ nanoparticles coadministration on the gastric genomic DNA integrity and inflammation induction was also estimated in the current study.

In contrast to the demonstrated Ca(OH)_2_ nanoparticles induced genotoxicity, oral coadministration of CaTiO_3_ and Y_2_O_3_ nanoparticles simultaneously with Ca(OH)_2_ nanoparticles was non-genotoxic and did not cause any noticeable changes in the integrity of gastric genomic DNA as detected by the non-significant changes observed in the measured DNA damage indicating parameters: tail length, %DNA in tail and tail moment after administration of Ca(OH)_2_, CaTiO_3_ and Y_2_O_3_ nanoparticles. Recently, Y_2_O_3_ nanoparticles showed antioxidant capacity through scavenging free radicals, thus administration of Y_2_O_3_ nanoparticles prohibits the induction of oxidative stress by decreasing the level of intracellular ROS generation and enhancing the antioxidant status of the cell^29,30^. Accordingly, the non-genotoxic effect detected after coadministration of CaTiO_3_ and Y_2_O_3_ nanoparticles simultaneously with Ca(OH)_2_ nanoparticles may result from the antioxidant and free radicals scavenging abilities of Y_2_O_3_ nanoparticles which prohibit Ca(OH)_2_ nanoparticles induced ROS generation and DNA damage.

Our findings of significant elevations in the gastric eNOS gene expression and non-noticeable changes in the gastric ROS level and expression level of inflammatory iNOS and COX-2 genes after coadministration of Ca(OH)_2_, CaTiO_3_ and Y_2_O_3_ nanoparticles revealed the absence of inflammation and oxidative stress and also confirmed the previously reported antioxidant and free radicals scavenging capacity of Y_2_O_3_ nanoparticles because gastric eNOS overexpression decreases the formation of ROS and increases the expression of antioxidants such as superoxide dismutase and Heme-oxygenase-I^22,29,30^.

## Conclusion

Based on the findings of this study, oral administration of Ca(OH)_2_ nanoparticles was genotoxic and disrupted the genomic DNA integrity through high ROS generation and overexpression of inflammatory iNOS and COX-2 genes. Conversely, oral administration of CaTiO_3_ and Y_2_O_3_ nanoparticles separately or together simultaneously with Ca(OH)_2_ nanoparticles was non-genotoxic and inhibited ROS overproduction and inflammation induction through upregulating eNOS gene expression that maintains on cell balance. Therefore, more in vivo and in vitro studies on the biological and toxic effects of Ca(OH)_2_, CaTiO_3_, or/and Y_2_O_3_ nanoparticles are recommended to understand their impact on human health.

## Data Availability

The datasets used and/or analyzed during the current study are available from the corresponding author on reasonable request.
